# The feasibility of CT simulation‐free adaptive radiation therapy

**DOI:** 10.1002/acm2.14438

**Published:** 2024-06-18

**Authors:** R. Lee MacDonald, Clara Fallone, Krista Chytyk‐Praznik, James Robar, Amanda Cherpak

**Affiliations:** ^1^ Department of Physics and Atmospheric Science Dalhousie University Halifax Nova Scotia Canada; ^2^ Department of Medical Physics Nova Scotia Health Queen Elizabeth II Health Sciences Centre Halifax Nova Scotia Canada; ^3^ Department of Radiation Oncology Dalhousie University Halifax Nova Scotia Canada

**Keywords:** adaptive, cone‐beam computed tomography, direct‐to‐unit

## Abstract

**Background:**

Novel on‐board CBCT allows for improved image quality and Hounsfield unit accuracy. When coupled with online adaptive tools, this may have potential to allow for simulation and treatment to be completed in a single on‐table session.

**Purpose:**

To study the feasibility of a high‐efficiency radiotherapy treatment workflow without the use of a separate session for simulation imaging. The dosimetric accuracy, overall efficiency, and technical feasibility were used to evaluate the clinical potential of CT simulation‐free adaptive radiotherapy.

**Methods:**

Varian's Ethos adaptive radiotherapy treatment platform was upgraded with a novel CBCT system, HyperSight which reports image quality and Hounsfield unit accuracy specifications comparable to standard fan‐beam CT. Using in‐house developed MATLAB software, CBCT images were imported into the system and used for planning. Two test cases were completed on anthropomorphic phantoms equipped with small volume ion chambers (cross‐calibrated to an ADCL traceable dose standard) to evaluate the feasibility and accuracy of the workflows. A simulated palliative spine treatment was planned with 8 Gy in one fraction, and an intact prostate treatment was planned with 60 Gy in 20 fractions. The CBCTs were acquired using HyperSight with default thorax and pelvis imaging protocols and reconstructed using an iterative algorithm with scatter removal, iCBCT Acuros. CBCTs were used for contouring and planning, and treatment was delivered via an online adaptive workflow. In addition, an external dosimetry audit was completed using only on‐board CBCT imaging in an end‐to‐end head and neck phantom irradiation.

**Results:**

An extended‐field CBCT acquisition can be acquired in 12 s, in addition to the time for longitudinal table shifts, and reconstructed in approximately 1 min. The superior‐inferior extent for the CBCT planning images was 38.2 cm, which captured the full extent of relevant anatomy. The contouring and treatment planning for the spine and prostate were completed in 30 and 18 min, respectively. The dosimetric agreement between ion chamber measurements and the treatment plan was within a range of −1.4 to 1.6%, and a mean and standard deviation of 0.41 ± 1.16%. All metrics used in the external audit met the passing criteria, and the dosimetric comparison between fan‐beam and CBCT techniques had a gamma passing rate of 99.0% with a criteria of 2%/2 mm.

**Conclusion:**

Using an in‐house workflow, CT simulation‐free radiation therapy was shown to be feasible with acceptable workflow efficiency and dosimetric accuracy. This approach may be particularly applicable for urgent palliative treatments. With the availability of software to enable this workflow, and the continued advancement of on‐treatment adaptation, single‐visit radiation therapy may replace current practice for some clinical indications.

## 1 INTRODUCTION

In the standard radiotherapy workflow, patients require imaging that simulates the treatment position prior to the start of treatment. This imaging appointment facilitates the long multi‐disciplinary process of contouring, planning, verifying, and approving treatment plans. These steps can take several hours, for urgent palliative treatments, up to several days or weeks, for more complex cases. With the advent of adaptive radiation therapy technology, standard radiation therapy workflows to generate a new treatment plan can be completed within approximately an hour of patient imaging.^1^

Recent development in cone‐beam computed tomography (CBCT) technology has allowed for treatment unit mounted imaging platforms which offer efficient high‐quality imaging suitable for dose calculation. The Varian (Varian Medical Systems, Siemens Healthineers, Erlangen, DE) HyperSight CBCT system implements this in imaging protocols with six‐second acquisition and accurate Hounsfield units (HU) with reference to fan‐beam CT acquisitions as baseline.^2^, ^3^, ^4^, ^5^, ^6^, ^7^ This enables the acquisition of on‐board images comparable to standard fan‐beam CT simulation during a standard treatment session. Examinations of the quality of anatomical delineation have also shown non‐inferiority between HyperSight and fan‐beam CT.^8^ Doses are also reduced with HyperSight imaging as compared to equivalent quality fan‐beam CT.^9^ When HyperSight high‐quality imaging is coupled with Varian's Ethos, a ring‐gantry linear accelerator with state‐of‐the art adaptive radiotherapy capabilities, the result may be a complimentary system for radiotherapy without simulation imaging. Additional groups have developed vendor‐agnostic CBCT reconstruction algorithms capable of HU‐accuracy, which may also encourage the use of direct calculation on CBCT in adaptive treatment settings.^10^

Recent publications have highlighted the efficiency advantages of single session radiotherapy in practice. The use of diagnostic imaging available prior to the decision to treat with radiation has been leveraged to inform same‐day palliative treatments.^8^, ^11^, ^12^, ^13^, ^14^, ^15^, ^16^ Due to inaccuracies in the HU of CBCT images, a synthetic‐CT is typically necessary. One method of creating a synthetic CT is to deformably register a previously acquired fan‐beam CT (FBCT) to represent the anatomy captured with a daily CBCT. The use of a synthetic‐CT allows for accurate dose calculation; however, it introduces potential uncertainty due to inaccurate deformation or inability to include densities found during an adaptive fraction that were initially not present during simulation (e.g., the presence or absence of bowel gas). Similar workflows have been demonstrated using MRI‐only protocols which make use of bulk‐density overrides or synthetic CT for dose calculation.^16^ This is implemented using MR‐guided radiotherapy platforms with adaptive radiotherapy technology. This protocol was applied in urgent palliation settings in which minimizing time to treatment is a clinical priority. Artificial intelligence (AI) based techniques have been implemented for interpreting CBCT information acquired during treatment to allow adaptive replanning without the use of simulation.^17^ This technique illustrated the reduction of department resources and time for mid‐treatment adaptation with simulation‐free replanning.

**FIGURE 1:**
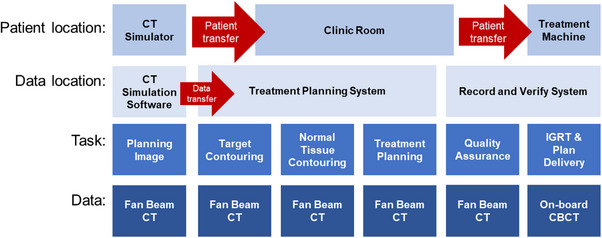
A standard workflow for palliative radiotherapy in which simulation and treatment occur in the same day. This appointment requires patient transfer, data transfer, and the use of multiple CT images.

**FIGURE 2:**
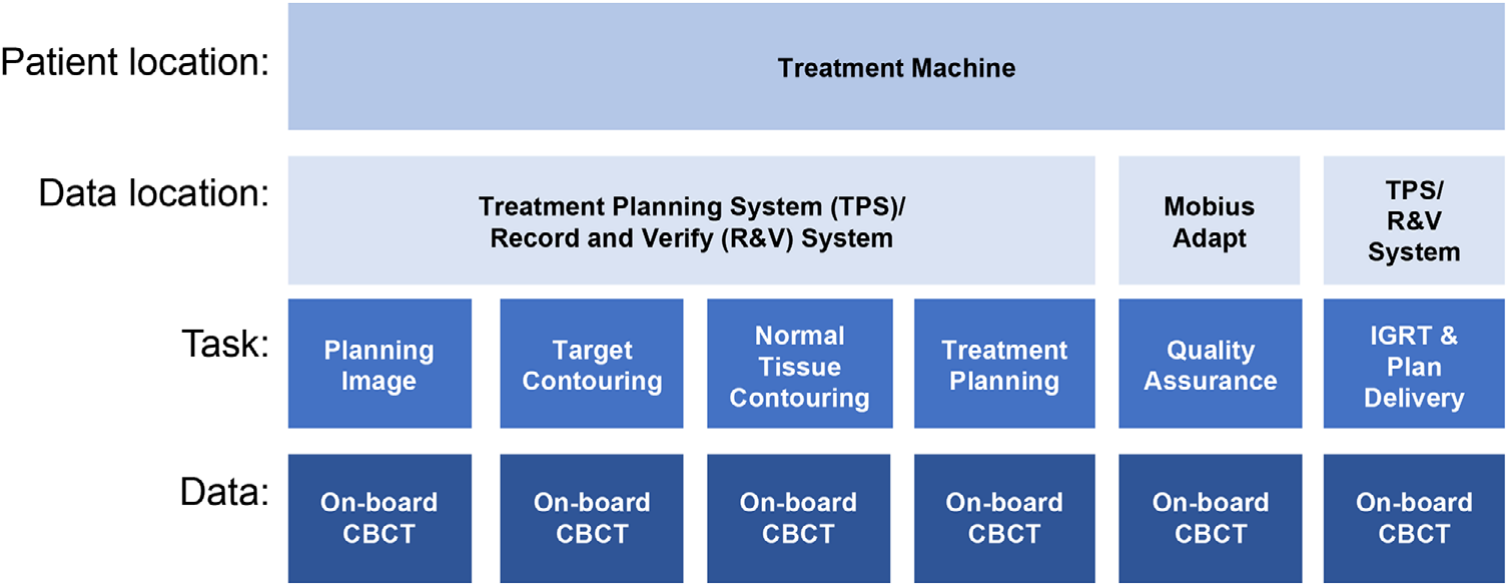
The proposed workflow which illustrates the efficiency in contrast to Figure 1. This workflow requires no patient transfer, uses only images acquired during the patient session, without data transfer, and does not remove any of the previous tasks. Mobius Adapt is Varian Medical System's independent monitor unit and dosimetric verification software.

To our knowledge, this work presents the first feasibility study on simulation‐free adaptive radiation therapy with dose calculation occurring solely on unmodified on‐board imaging from the same on‐couch session. While previous work has illustrated the dose calculation accuracy component of this workflow, this study focusses on the end‐to‐end simulation‐free workflow.^9^ The workflow for standard palliative radiotherapy as it is currently implemented clinically is shown in Figure 1. Figure 2 shows the proposed workflow, in which the patient arrives directly to the treatment unit for simulation, contouring, planning, quality assurance, and delivery. While presently, our institutional quality assurance is deployed and analyzed within the record and verify system (ARIA Portal Dose verification), an independent monitor unit calculation check is conducted for the novel workflow in Figure 2. This step may involve the use of additional platforms, depending upon the procedures utilized to complete pre‐treatment quality assurance.

## 2 METHODS

To assess the feasibility of performing adaptive radiation therapy without simulation imaging, three different experiments were conducted using exclusively on‐board CBCT imaging for planning and treatment. The first was a proof‐of‐concept end‐to‐end test using an anthropomorphic spine phantom. This was to assess whether the system could allow the technical steps of this workflow. Secondly, an adaptive male pelvis phantom was used in an end‐to‐end test complete with ion chamber measurement. This phantom was used to assess the dosimetric agreement between planned and delivered doses, in both standard and adaptive planning strategies. Finally, an independent dosimetric audit was completed using a phantom equipped with radiochromic film and thermo‐luminescent dosimeters (TLD). The Imaging and Radiation Oncology Core (IROC) quality assurance center at MD Anderson provided this external auditing of dosimetry, typically intended for applications such as credentialling in radiation therapy clinical trials and internal verification of output dosimetry or new techniques.

### 2.1 Adaptive spine treatment

**FIGURE 3:**
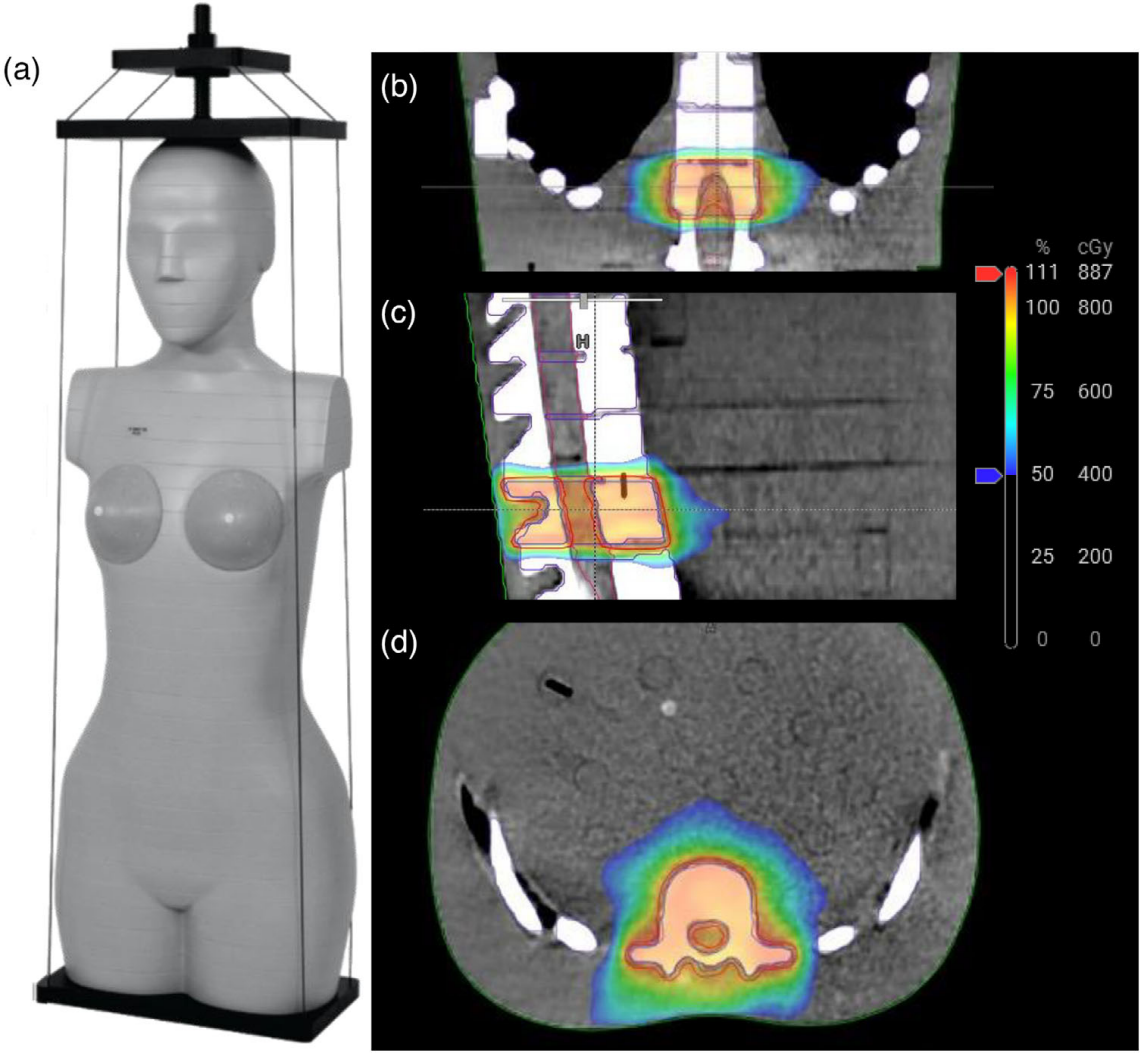
(a) The Sun Nuclear ATOM series phantom used for this experiment. (b–d) Coronal, sagittal, and axial views of the 8 Gy in one dose distribution for the spine phantom testing, respectively.

The SunNuclear (Sun Nuclear Corporation, Melbourne, FL, USA) ATOM series adult female anthropomorphic phantom was imaged in Head‐First‐Supine position on an Ethos unit equipped with HyperSight (Figure 3a). As the first step in the process, an extended CBCT was acquired using two standard acquisitions combined after a shift in the superior‐inferior direction. The scanning protocol was the default standard thorax (125 kV, 174 mAs, 2 mm slice thickness). The total superior‐inferior extent of the image was 38.2 cm. The field of view (FOV) in the reconstruction was 53.8 cm. The reconstruction algorithm used was iCBCT Acuros which includes novel algorithms allowing improved image quality and HU accuracy (and used for all reconstructions in this work). This image set was imported to Ethos as the reference scan for offline treatment planning.

All contouring, planning, and approvals were completed in Ethos Treatment Management (Varian Medical Systems). In‐house MATLAB software was developed to modify the necessary DICOM tags for this treatment planning system to interpret this image set as usable for planning. The phantom was contoured for a simulated palliative spine treatment of 8 Gy delivered in 1 fraction. A single vertebral body was contoured as a GTV, and a 5 mm expansion was used to create the PTV. The axial planes of the target included heterogeneous media such as simulated lung, bone, and soft tissue. A two‐arc VMAT adaptive plan was automatically generated with 90% of the prescription dose covering 99% of our target volume as outlined in the clinical protocol for palliative treatment (Figure 3b–d). VMAT was selected as the treatment technique for the work in this manuscript as it was the clinically implemented technique at the time of this experiment. This offline plan generated using the initial image is referred to in Ethos as the Reference plan. The two online plans are termed the Scheduled plan (the reference plan calculated on the daily image and contours), and the adapted plan (the adapted plan reoptimized/calculated on the daily image and contours). Independent planned calculation‐based dosimetry verification was completed using Mobius3D (Varian Medical Systems). Treatment was delivered on the Varian Ethos system using an adaptive workflow in which the phantom was imaged a second time, which was used as the adaptive planning image. Verification of delivery was completed using Mobius to reconstruct dose based on log‐file analysis.

### 2.2 Standard prostate treatment with matched anatomy

**FIGURE 4:**
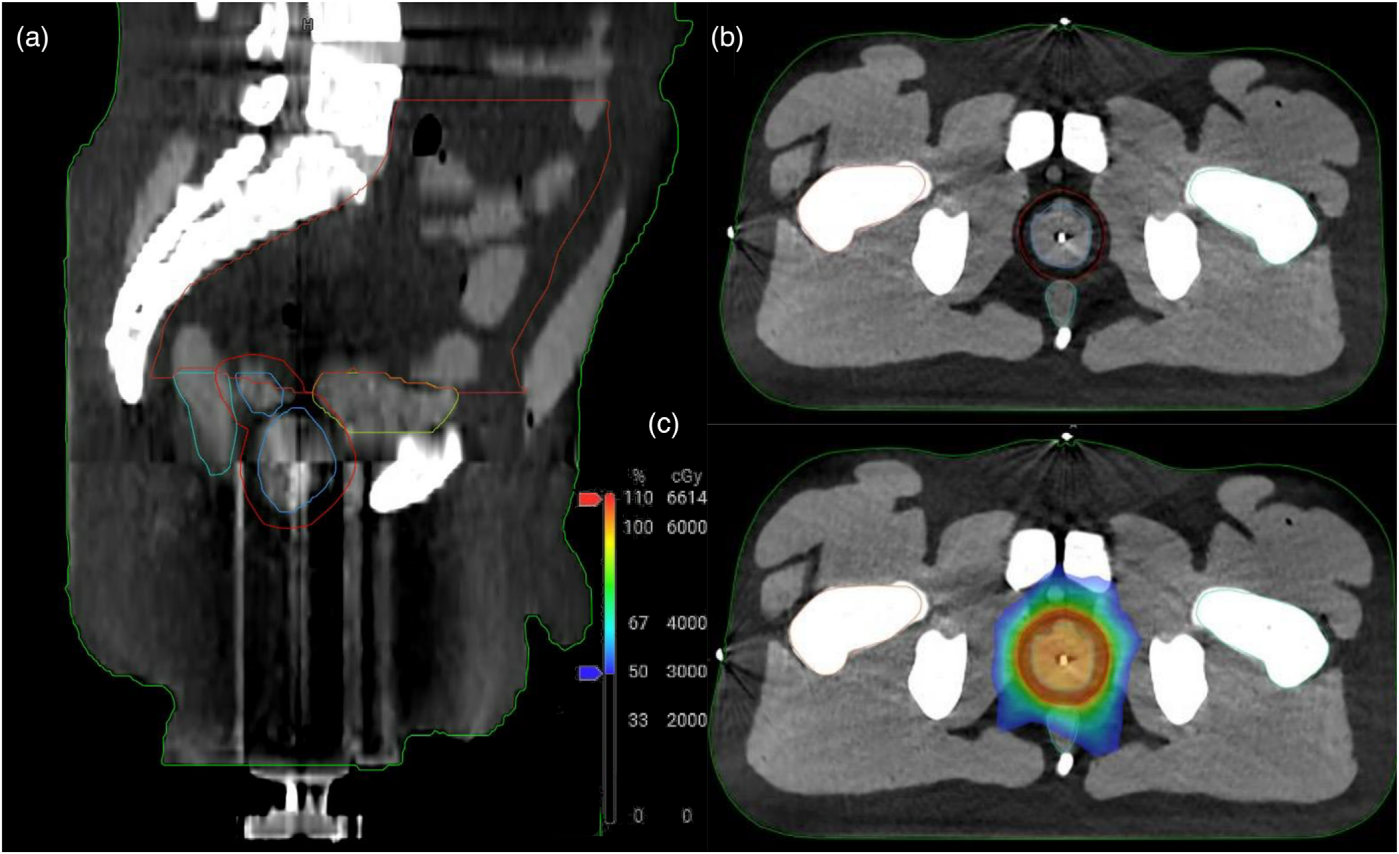
(a, b) Orthogonal views of contoured structures used to generate reference and adapted plans. Imaging was acquired using Varian HyperSight CBCT. (c) Axial view of the dose distribution for the 60 Gy in 20 fraction plan generated in Ethos. The displayed contours are: CTV (blue), PTV (red), bowel bag (orange), bladder (yellow), rectum (teal), external body (green).

An anthropomorphic male pelvis phantom manufactured by CIRS (Sun Nuclear Corporation, Melbourne, FL, USA) (Figure 4) was modified by the manufacturer with a cavity and insert containing a prostate volume, and a channel to house an ion chamber. Two inserts were available, to introduce either a 15 cm^3^ or a 25 cm^3^ prostate volume. In addition, there was a second channel to house an ion chamber in the bladder. The phantom was imaged with the larger prostate size using a HyperSight extended CBCT with the same physical scan dimensions as above; however, using the default pelvis protocol (125 kV, 468 mAs, 2 mm slice thickness). This image was used as the reference image set for planning. An experienced radiation therapist completed the contouring of the phantom for all target volumes, and organs‐at‐risk (OARs). The ion chamber cavities were contoured in the reference scan to use for dose tracking. A two‐arc VMAT prostate plan was generated to deliver 60 Gy in 20 fractions to the prostate according to institutional protocol. The phantom was set up using the same insert as the reference scan, and a standard (non‐adaptive) fraction was delivered using the scheduled reference plan with image guidance. IBA (IBA International, Louvain‐La‐Neuve, BE) CC13 small volume ion chambers were used in the phantom channels, having been cross calibrated with traceable accredited dosimetry calibration laboratory (ADCL) results. Independent planned dosimetry verification was completed using Mobius3D. Treatment was delivered on the Varian Ethos system, and verification of delivery was completed using Mobius. A comparison of doses delivered to the ion chamber was made with treatment planning system doses to assess the agreement of planned and delivered doses.

### 2.3 Standard and adaptive prostate treatments with modified anatomy

The same methodology as described in Section 2.2 was used, with the smaller prostate (15 cm^3^) insert in the phantom. Two separate treatment fractions were completed: one standard (non‐adaptive) fraction using the reference plan, and an adaptive fraction. In both sessions, measured dose values delivered to each ion chamber were compared with treatment planning system reported doses. Independent planned dosimetry verification was completed using Mobius3D. Treatment was delivered on the Varian Ethos system, and verification of delivery was completed using Mobius.

### 2.4 External dosimetric audit

The IROC head and neck (H&N) phantom is intended for confirmation of radiotherapy using high‐quality IMRT.^18^ The protocol stipulates doses to internal target and normal tissue structures. The phantom is equipped with TLD and film dosimeters which evaluate absolute and relative dose, respectively, as well as the spatial agreement of planned and delivered doses. The IROC H&N phantom protocol was completed using exclusively HyperSight images. The contouring and treatment planning was completed in Ethos. Independent plan dosimetry verification was completed using Mobius3D. Treatment was delivered on the Varian Ethos system with verification of delivery completed using Mobius.

## 3 RESULTS

### 3.1 Adaptive spine treatment

The full end‐to‐end adaptive workflow on Ethos was completed using the HyperSight imaging as the reference image. Comparison of dosimetry between the HyperSight calculated distribution and the same plan forward calculated on an FBCT showed excellent dosimetric agreement, passing a 3%/3 mm, 2%/2 mm, and 1%/1 mm gamma comparison for 100%, 99.0%, and 94.2% of sampled points, respectively. The adaptive fraction, including reference scan and initial contouring, was completed in 33 min. The independent monitor unit verification software Mobius reported a 99.9% gamma pass rate with criteria of 3%/3 mm. The agreement between reconstructed dose based on the log‐file resulted in a gamma pass rate of 100% at 3%/3 mm.

### 3.2 Standard prostate treatment with matched anatomy

**FIGURE 5:**
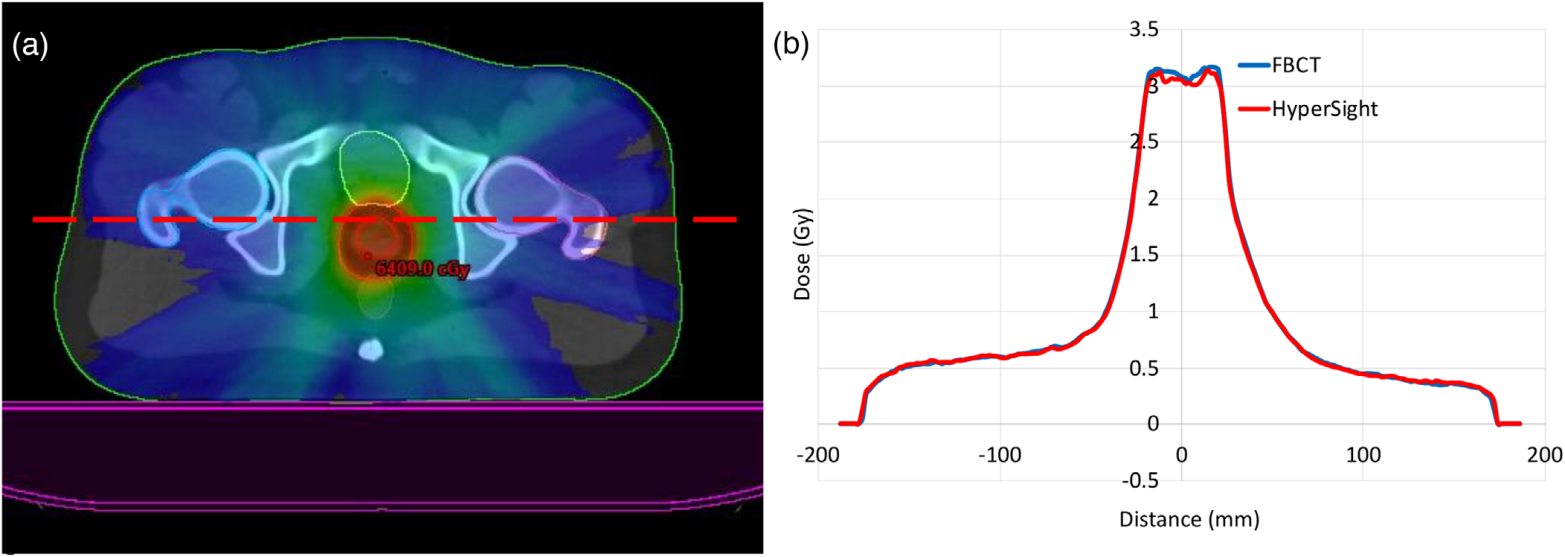
Dose profiles through the same plan calculated on HyperSight and fan beam CT (FBCT) images. (a) shows the dose distribution on the FBCT with the location of the dose profile shown at the red dotted line. (b) Shows the dose profiles as measured using PTW Verisoft with the red profile for HyperSight and the blue profile for FBCT. The gamma was measured in PTW Verisoft for 3%/3 mm, 2%/2 mm, and 1%/1 mm with passing rates of 100%, 99.0%, and 94.2% respectively.

**FIGURE 6:**
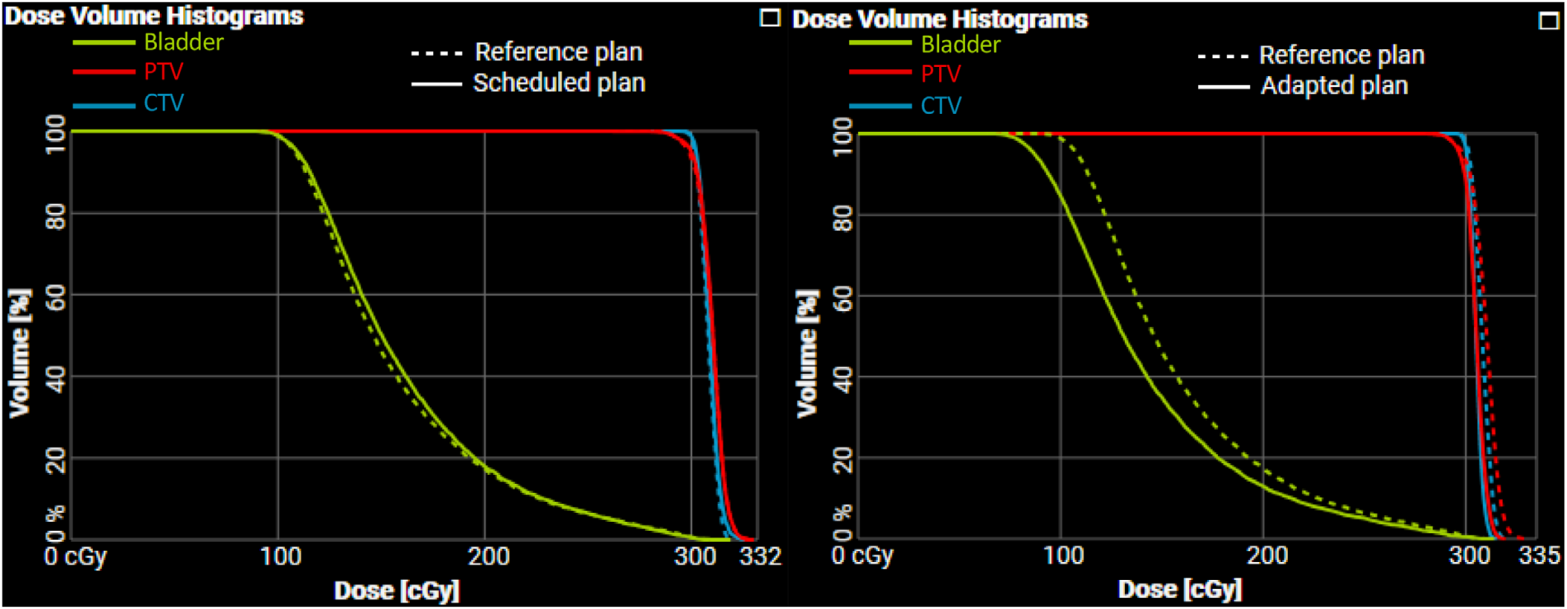
(a) Results of a standard (non‐adaptive) fraction completed using the same prostate target size (25 cm^3^) as used in planning. (b) Results of an adaptive fraction delivered to the smaller (15 cm^3^) prostate target than that used in planning. Table 1 shows ion chamber measurements reflecting the differences seen in the reported adaptive doses.

A standard (non‐adaptive) end‐to‐end fraction was completed using the prostate phantom with the same 25 cm^3^ prostate insert that was used during acquisition of the reference CBCT. The DVH for the reference and schedule plan is shown in Figure 6(a) for the CTV, PTV, and Bladder volumes. The tabulated ion chamber dose readings are shown in Table 1. A comparison of measured values and treatment planning system reported mean doses for the contoured active volume of the CC13 ion chamber show agreement within 1.5%. The fraction was completed in 34 min including treatment delivery. The independent monitor unit verification software Mobius reported a 99.7% gamma pass rate with criteria of 3% / 3 mm (the criteria used clinically for dose verifications at our institution). The agreement between reconstructed dose based on the log‐file resulted in a gamma pass rate of 100% at 3% / 3 mm. Figure 5(b) shows a dose profile through the plan dose calculated on both fan‐beam CT and HyperSight CT at the location indicated by Figure 5(a). The gamma pass rate was measured in PTW Verisoft for criteria 3%/3 mm, 2%/2 mm, and 1%/1 mm with passing rates of 100%, 99.0%, and 94.2%, respectively.

**TABLE 1:** A comparison of CC13 ion chamber doses (measured) compared to TPS reported doses (planned) for three different treatment plans and scenarios.

	Chamber position	Planned (cGy)	Measured (cGy)	Difference (%)
Matched anatomy non‐adaptive	Bladder	176.47	178.98	+1.4
	Prostate	315.84	312.16	−1.2
Modified anatomy non‐adaptive	Bladder	173.45	176.35	+1.6
	Prostate	314.18	312.43	−0.6
Modified anatomy adaptive	Bladder	156.74	154.87	+1.2
	Prostate	309.95	309.99	0.0

*Note*: The difference is calculated via percentage difference.

### 3.3 Standard and adaptive prostate treatment with modified anatomy

With the smaller (15 cm^3^) insert in the prostate phantom, both an adaptive and non‐adaptive (standard) fraction were completed. A comparison of ion chamber measurement and treatment planning system doses can be seen with agreement within 1.6%. The DVHs for the CTV, PTV and bladder can be seen in Figure 6. The yellow curve indicates the DVH for the bladder, which is markedly improved in the adaptive fraction, indicating a change in dosimetry was made during adaptation. The fraction was completed for the standard and adaptive treatments in 33 and 34 min, respectively. The independent monitor unit verification software Mobius reported a gamma pass rate of 99.9% and 100% for the standard and adaptive fractions, respectively, with gamma criteria of 3%/3 mm. The agreement between reconstructed dose based on the log‐file resulted in a gamma pass rate of 100% and 100% at 3%/3 mm for the standard and adaptive fractions, respectively. The cumulative time for each fraction as well as the time to complete each task is shown in Table 2.

**TABLE 2:** Recorded time for each task required for the workflow in the prostate phantom workflow as recorded in the Ethos planning system.

Delivered plan	Simulation imaging	Contouring and planning	Adaptive contouring and assessment	Delivery	Total time on bed
Prostate matched scheduled plan	2 min	18 min	15 min	2 min	34 min
Prostate modified scheduled plan	–	–	11 min	2 min	33 min
Prostate modified adapted plan	–	–	12 min	3 min	35 min

### 3.4 External dosimetry audit

The protocol was completed using the procedures from IROC accompanying the Head and Neck phantom. Table 3 illustrates the ratios of treatment planning system reported mean doses, and the corresponding readings on the TLDs. All results met the passing criteria for the Head and Neck phantom protocol, using exclusively HyperSight imaging for the end‐to‐end procedure. The planning image set was uploaded in DICOM format and required no modification to comply with IROC evaluation.

**TABLE 3:** Comparison between treatment planning system reported doses and IROC reported TLD readings.

Location	Ethos reported mean dose (cGy)	IROC TLD dose (cGy)	Ratio of IROC to institution
Primary PTV superior anterior	684	699	1.02
Primary PTV inferior anterior	691	709	1.03
Primary PTV superior posterior	686	699	1.02
Primary PTV inferior posterior	692	708	1.02
Secondary PTV superior	561	563	1.00
Secondary PTV inferior	565	562	0.99
Organ‐at‐risk superior	179	182	1.02
Organ‐at‐risk inferior	180	186	1.03

*Note*: All values satisfy the agreement criteria to pass the external dosimetry audit.

## 4 DISCUSSION

Radiotherapy that is planned using imaging acquired in the same session as treatment has advantages that are threefold: the first is a reduction in time and resources for the department. Removing the need for simulation imaging reduces the number of visits required for the patient, and the personnel, equipment, and time to acquire simulation imaging. The second is an ability to rapidly deliver palliative treatment in urgent settings. It is of high priority to rapidly respond to urgent need for palliative radiation therapy, and a workflow which can complete the entire radiotherapy process in under an hour would be a significant improvement in this objective. Finally, having the treatment created and adapted to anatomical positioning immediately prior to treatment ought to improve the precision of radiation delivery. While this is presently achievable, it relies on a synthetic CT. Relying solely on the in‐sessions images reduces uncertainty due to mitigating uncertainty associated with synthetic CT generation (i.e., deformable image registration). In an IGRT workflow, an alignment of the patient is made to mitigate discrepancies in positioning prior to delivery. Inevitably, there is some residual discrepancy, which may be reduced with the use of single‐session radiotherapy.

While the workflows presented in this manuscript create a reference plan using specific reference images as a basis for the adaptive fraction, it is likely that with the advancement of adaptive technologies, this could be replaced with a library of base plans that can be adapted to patient imaging. This would reduce the number of images required at the unit to complete the proposed workflow and improve overall efficiency.

Reviewing the results displayed in this work, we found excellent dosimetric agreement between plans generated in single‐session radiotherapy using exclusively on‐treatment images and traditionally calculated plans. In spine, prostate, and head and neck planning, the completed workflow was feasible within clinically realistic timeframes. Both standard and adaptive fractions showed good agreement with ion chamber measured doses. In the adaptive fraction, the reduction of CTV volume allowed the dose to the bladder to be reduced as reported by the planning system. This reduction is reflected in the values in Table 1. An important limitation of this study is that the phantoms do not mimic movement over the fraction timeline. While the prostate phantom was able to simulate a significant change in CTV volume, deformable changes in external body contour or internal structures occurring intrafractionally (e.g., breathing motion) were not able to be simulated and there are potential uncertainties introduced if the 6‐s scan time does not capture all relevant motion. Also, as VMAT was the only treatment technique used in this manuscript, there may be efficiency differences between IMRT and VMAT treatment calculation which might further reduce the overall workflow time (due to decreased plan generation time).

The images acquired at the beginning of each session were chosen as an extended CBCT to ensure sufficient superior‐inferior extent required for contouring and dose calculation. An audit of recently treated single‐site palliative CT simulation scans had an average length of 44.9 cm, and the extended CBCT has a length of 38.2 cm. This indicates that HyperSight extended CBCT can capture a comparable extent of anatomy as is done using current FBCT clinical protocols for CT‐Simulation. Depending on disease site, a standard CBCT field of view may suffice, further reducing the overall time for this workflow.

## 5 CONCLUSION

Using these clinical tools and methods, CT simulation‐free radiation therapy was shown to be feasible without compromise in dosimetry compared to standard fan‐beam simulation. This approach has the potential for use in urgent palliative treatments in which minimizing the time between decision to treat, and treatment is a clinical priority. With the improvement of software to allow this workflow without modification to DICOM header information, and the continued advancement of on‐treatment adaptation, single‐visit radiation therapy may soon replace current practice for some clinical indications.

## AUTHOR CONTRIBUTIONS

**R. Lee MacDonald**: Manuscript preparation; research formation; methodology contribution. **Clara Fallone**: Methodology contribution; manuscript contribution. **Krista Chytyk‐Praznik**: Methodology contribution; manuscript contribution. **James Robar**: Methodology contribution; manuscript contribution. **Amanda Cherpak**: Methodology contribution; manuscript contribution.

## CONFLICT OF INTEREST STATEMENT

The authors declare no conflicts of interest.
